# Deep learning for transesophageal echocardiography view classification

**DOI:** 10.1038/s41598-023-50735-8

**Published:** 2024-01-02

**Authors:** Kirsten R. Steffner, Matthew Christensen, George Gill, Michael Bowdish, Justin Rhee, Abirami Kumaresan, Bryan He, James Zou, David Ouyang

**Affiliations:** 1 Department of Anesthesiology, Perioperative and Pain Medicine, Stanford University, 300 Pasteur Drive, Stanford, CA 94305 USA; 2 Department of Cardiology, Smidt Heart Institute, Cedars-Sinai Medical Center, Los Angeles, USA; 3 Department of Cardiac Surgery, Smidt Heart Institute, Cedars-Sinai Medical Center, Los Angeles, USA; 4 Department of Anesthesiology, Cedars-Sinai Medical Center, Los Angeles, USA; 5 Department of Computer Science, Stanford University, Stanford, USA; 6 Department of Biomedical Data Science, Stanford University, Stanford, USA

**Keywords:** Cardiology, Machine learning

## Abstract

Transesophageal echocardiography (TEE) imaging is a vital tool used in the evaluation of complex cardiac pathology and the management of cardiac surgery patients. A key limitation to the application of deep learning strategies to intraoperative and intraprocedural TEE data is the complexity and unstructured nature of these images. In the present study, we developed a deep learning-based, multi-category TEE view classification model that can be used to add structure to intraoperative and intraprocedural TEE imaging data. More specifically, we trained a convolutional neural network (CNN) to predict standardized TEE views using labeled intraoperative and intraprocedural TEE videos from Cedars-Sinai Medical Center (CSMC). We externally validated our model on intraoperative TEE videos from Stanford University Medical Center (SUMC). Accuracy of our model was high across all labeled views. The highest performance was achieved for the Trans-Gastric Left Ventricular Short Axis View (area under the receiver operating curve [AUC] = 0.971 at CSMC, 0.957 at SUMC), the Mid-Esophageal Long Axis View (AUC = 0.954 at CSMC, 0.905 at SUMC), the Mid-Esophageal Aortic Valve Short Axis View (AUC = 0.946 at CSMC, 0.898 at SUMC), and the Mid-Esophageal 4-Chamber View (AUC = 0.939 at CSMC, 0.902 at SUMC). Ultimately, we demonstrate that our deep learning model can accurately classify standardized TEE views, which will facilitate further downstream deep learning analyses for intraoperative and intraprocedural TEE imaging.

## Introduction

Cardiovascular disease is a leading cause of death and disability worldwide and has been one of the top ten most important drivers of increasing global disease burden in the last three decades^1^. Echocardiography is the most commonly used imaging modality in the assessment of cardiac structure, function, and disease^2,3^. The two main modalities of echocardiography imaging are transthoracic echocardiography (TTE) and transesophageal echocardiography (TEE). TTE imaging is used as a screening tool in asymptomatic patients and as the initial diagnostic tool for many cardiovascular disease states, including ischemic heart disease, valvular heart disease, rhythm disorders, and heart failure. TEE imaging is utilized in the workup and management of complex cardiac pathology such as sequelae after acute myocardial ischemia or acute aortic disease^3^. TEE is particularly valuable as a monitoring and diagnostic tool utilized in the management of cardiac surgery patients^2,4^. As the standard of care, intraoperative TEE imaging is performed during all major cardiac surgeries, especially those requiring an open sternotomy and cardiopulmonary bypass (CPB), to help make diagnoses, guide surgical decision-making, and evaluate hemodynamic states in real-time.

Given its importance in cardiovascular disease management, echocardiography imaging has become an important target for artificial intelligence (AI). Prior echocardiography-based AI research has focused on TTE videos, with recent work showing that machine learning algorithms are able to classify standardized TTE views^5–8^, recognize cardiac structures^9^, and estimate left ventricular ejection fraction^10^. Additional work has demonstrated the ability to accurately diagnose the etiology of left ventricular hypertrophy^11^, extract phenotypic information such as age and sex^9^, and predict clinical outcomes such as postoperative right ventricular failure after the implantation of a left ventricular assist device^12^.

Previous groups have also shown that machine learning models can be trained on TEE data to perform focused image segmentation tasks and automatically calculate measurements such as the mitral annular plane systolic excursion; however, such TEE-based approaches have been limited to small and highly-curated data sets^13–15^. The application of AI and machine learning to TEE images acquired during the course of standard clinical care remains relatively unexplored. TEE imaging data is highly variable due to the dynamic environment in the cardiac surgery operating rooms, which results in the acquisition of varying image sequences, non-standard views, and missing views. Without an automated preprocessing and view classification pipeline for clinically-acquired TEE videos, deep learning tasks on unstructured intraoperative and intraprocedural TEE data remains challenging.

However, given the vitally important role that TEE imaging plays in the evaluation of complex cardiovascular disease states and in the perioperative management of high-risk cardiac surgery patients, there is great potential value to be extracted from TEE images with advanced deep learning methodologies. Therefore, the purpose of the present study was to train a deep learning-based TEE view classification model that could be used to create structure for intraoperative and intraprocedural TEE imaging data and thereby facilitate downstream TEE-based deep learning tasks. More specifically, the aim of the present study was to train a convolutional neural network (CNN) to accurately classify standardized TEE views using labeled intraoperative and intraprocedural TEE videos.

## Methods

### Cohort selection and data processing

We obtained TEE image data for randomly selected adult patients who underwent an intraoperative or intraprocedural TEE exam at Cedars-Sinai Medical Center (CSMC) between the years of 2016 and 2021. This resulted in 2967 TEE videos, including intraoperative echocardiography images from open (via sternotomy) cardiothoracic surgical operations and intraprocedural echocardiography images from transcatheter procedures for structural heart disease. We also obtained TEE image data from randomly selected adult patients who underwent an intraoperative TEE exam during open cardiothoracic surgery at Stanford University Medical Center (SUMC), resulting in an additional 465 TEE videos for an external test set.

The Institutional Review Board at Cedars-Sinai Medical Center and the Institutional Review Board at Stanford University Medical Center both granted ethical approval for this study. Given the nature of our study as a retrospective analysis of data that had already been collected as part of the clinical standard of care, a waiver of informed consent was granted by the Institutional Review Boards at both Cedars-Sinai Medical Center and Stanford University Medical Center. All study methods were performed in accordance with the guidelines and regulations outlined by both Institutional Review Boards.

TEE image data was converted from Digital Imaging and Communications in Medicine (DICOM) format data to AVI videos. Prior to labeling, model training, and analysis, an automated preprocessing workflow was undertaken to remove patient identifying information and eliminate unintended human labels. Each subsequent video was cropped and masked to remove text, ECG and respirometer information, and other information outside of the scanning sector. The resulting square images were either 600 × 600 or 768 × 768 pixels depending on the ultrasound machine and down-sampled by cubic interpolation using OpenCV into standardized 112 × 112 pixel videos.

All training, validation, and test images were labeled by a board-certified echocardiographer. Expert consensus echocardiography guidelines identify twenty-eight standardized TEE views for a complete intraoperative multi-plane TEE exam^16^. For our multi-category deep learning view classification model, we chose the eight most consistently acquired and most clinically useful TEE views in the intraoperative assessment of cardiac surgery patients, including: the Mid-Esophageal (ME) 2-Chamber View, ME 4-Chamber View, ME Aortic Valve (AV) Short Axis (SAX) View, ME Bicaval View, ME Left Atrial Appendage View, ME Long Axis View, Trans-Gastric (TG) LV SAX View, and Aortic View.

Four of our eight chosen views (the ME 2-Chamber, ME 4-Chamber, ME AV SAX, ME Long Axis) represent pooled categories that integrate two of the twenty-eight standardized views. More specifically, the “ME 2-Chamber View” class includes ME 2-chamber and ME mitral commissural views; the “ME 4-Chamber View” class includes ME 4-chamber and ME 5-chamber views; the “ME AV SAX View” class includes ME AV SAX and ME right ventricular (RV) inflow-outflow views; and the “ME Long Axis View” class includes ME long axis and ME AV long axis views. We also chose to generalize two categories (the TG LV SAX and the Aortic Views). TEE videos that did not fall into any of the eight chosen view classes were labeled as “Other.”

### AI model design and testing

We trained a CNN to classify eight standardized TEE views. Our training and validation sets contained 2464 unique videos (split 4:1), representing 2036 patients. The model was tested on 503 randomly selected videos from CSMC and 465 randomly selected videos from SUMC, none of which were seen during model training. We trained the CNN with residual connections and spatiotemporal convolutions using the R2 + 1D architecture^17,18^. We chose R2 + 1D spatiotemporal convolutions based on our prior work with TTE videos, where we tested multiple model architectures with variable integration of temporal convolutions and found decomposed R2 + 1D spatiotemporal convolutions to have the best balance of computational complexity and model performance^10^. A further description of model architecture and tradeoffs are well described in the original architecture papers^17,18^.

Model weights were randomly initialized. Models were trained to minimize the cross entropy between the predicted view and the actual labeled view. We used an Adam optimizer^19^, a learning rate of 0.001, and a batch size of 44. We employed early stopping to cease model training after no further improvement on the validation set occurred. Our final model trained for nine epochs. The model was trained on 32-frame sub-clips of videos in the training set, with a temporal stride of two, yielding a final model input length of 16 frames. The choice of 16 frames is based on hyperparameter sweeps in prior work balancing model performance and computational efficiency^10^. The starting frame of these sub-clips within their parent clips were randomized during training as a form of data augmentation. All model training was done using the Python library PyTorch. Our code is available online at https://github.com/echonet/tee-view-classifier.

### Statistical analysis

An internal hold-out test data set from CSMC which was never seen during model training was used to assess model performance. An external test set from SUMC was also used for additional testing and was never seen during model training. Model performance was assessed via AUROC. Two-sided 95% confidence intervals using 1000 bootstrapped samples were computed for each calculation. Unsupervised t-Distributed Stochastic Neighbor Embedding (t-SNE) was used for clustering analysis^20^. All statistical analyses were performed in Python.

## Results

**Figure 1:**
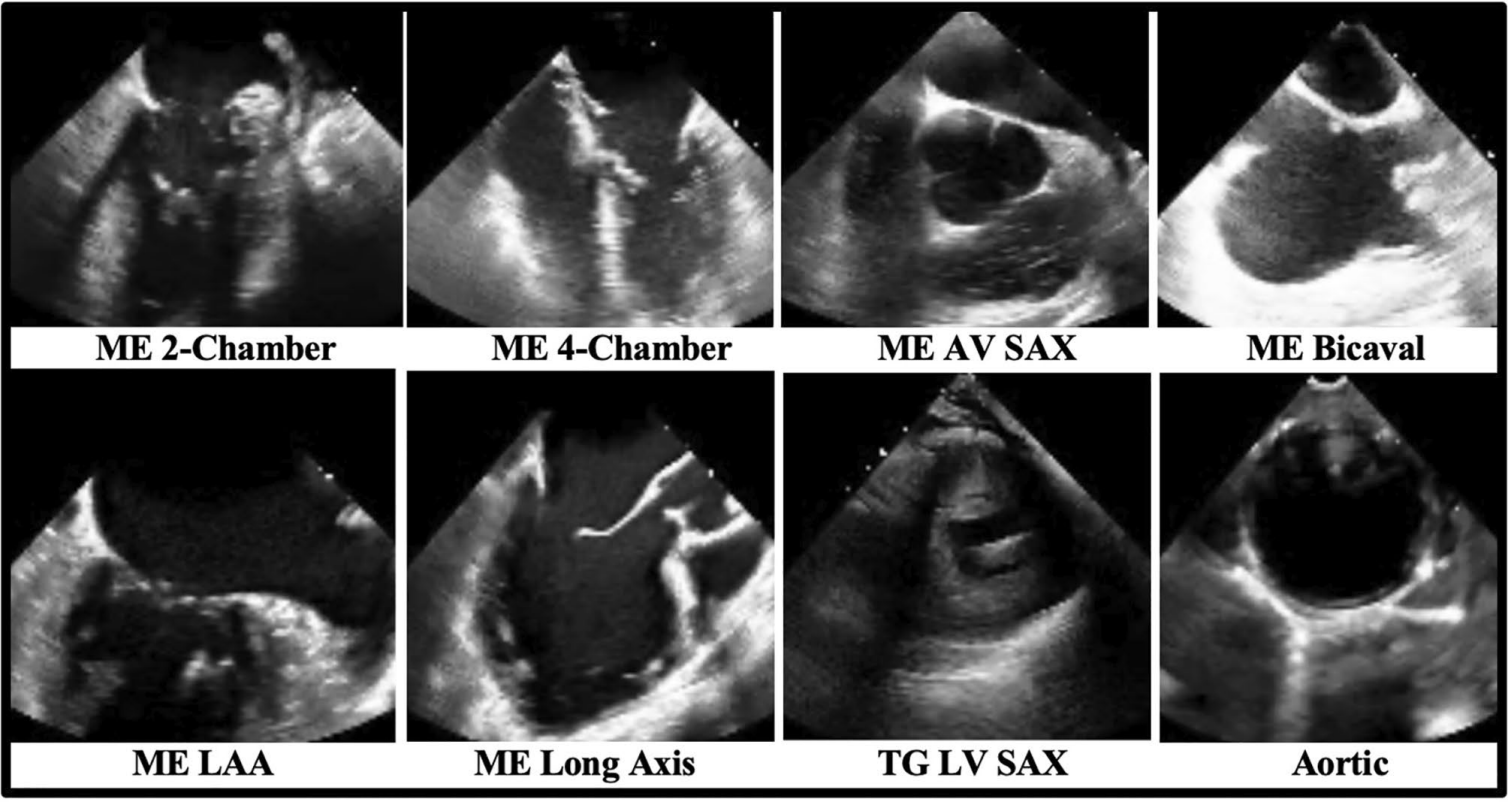
Sample training images used for the deep-learning view classification task. Images are 2-dimensional still frames sampled from the video data used in model training. Eight standard TEE views were chosen, including: the ME 2-Chamber View, ME 4-Chamber View, ME AV SAX View, ME Bicaval View, ME LAA View, ME Long Axis View, TG LV SAX View, and Aortic View. TEE, transesophageal echocardiography; ME, mid-esophageal; AV, aortic valve; SAX, short axis; LAA, left atrial appendage; TG, trans-gastric; LV, left ventricular.

Patient characteristics and surgery or procedure types represented in our training, validation, and test data sets are shown in Table 1. Our data sets included a broad spectrum of anatomic variation, clinical pathology, and imaging indications reflecting the cardiac open surgical and transcatheter procedural populations seen at CSMC and SUMC. The images also included a wide range of technical variation, including differences in spatial and temporal resolution, field of view depth and sector width, gain, image quality, and use of color flow Doppler (Fig. 1). The most frequently represented views included the ME-4 Chamber View, the ME Long Axis View, the TG Left Ventricular Short Axis View, and the ME Aortic Valve Short Axis View (Table 2).

**Table 1:** Clinical characteristics and surgery or procedure types represented in the training, validation, and internal test data sets.

	Total	Train	Validation	Internal Test
Number of videos (n)	2967	1968	496	503
Mean age, years (SD)	67.8 (± 15.4)	67.9 (± 15.4)	68.3 (± 15.7)	66.9 (± 15.2)
White (%)	71.6%	72.1%	69.7%	72.0%
Black (%)	10.2%	10.8%	11.8%	6.2%
Other/unknown (%)	8.7%	8.5%	7.8%	10.5%
Asian (%)	7.8%	7.3%	7.6%	9.9%
Pacific Islander (%)	1.1%	0.9%	2.7%	0.6%
Native American (%)	0.4%	0.3%	0.4%	0.8%
Hispanic ethnicity (%)	12.7%	12.3%	13.0%	14.3%
Female gender (%)	36.1%	36.5%	37.0%	33.6%
Atrial fibrillation (%)	36.9%	37.2%	37.0%	35.6%
Heart failure (%)	48.5%	48.6%	50.2%	46.1%
Hypertension (%)	54.2%	55.0%	51.0%	54.1%
Diabetes mellitus (%)	22.6%	23.3%	21.4%	21.3%
Ischemic stroke (%)	11.8%	12.3%	10.1%	11.3%
Transient ischemic attack (%)	6.3%	6.6%	5.9%	5.2%
Pulmonary embolism (%)	2.9%	3.0%	4.0%	1.2%
Myocardial infarction (%)	10.9%	10.3%	10.5%	13.9%
Peripheral artery disease (%)	17.4%	18.1%	15.8%	16.1%
Vascular disease (%)	26.2%	26.5%	24.0%	27.2%
Coronary artery disease (%)	37.1%	37.0%	36.6%	38.0%
Chronic kidney disease (%)	24.0%	23.8%	26.7%	21.9%
Liver disease (%)	5.1%	5.3%	4.6%	5.0%
Chronic obstructive pulmonary disease (%)	6.4%	6.7%	5.2%	6.8%
Prior smoker (%)	5.8%	5.7%	6.7%	5.4%
CABG (%)	6.3%	6.0%	5.9%	7.8%
Valve procedure (%)	10.3%	10.4%	8.0%	12.7%
Aortic procedure (%)	1.4%	1.4%	0.6%	1.8%
Combination of CABG, valve, and/or aortic procedure (%)	7.0%	6.7%	6.7%	8.5%
Other open cardiac procedure (%)	10.2%	10.4%	10.7%	9.1%
Mechanical circulatory support (%)	1.7%	1.4%	2.5%	1.8%
Transcatheter procedure (%)	63.1%	63.7%	65.6%	58.3%

SD, standard deviation. CABG, coronary artery bypass graft.

**Table 2:** Number of TEE videos labeled for model training and validation, per view class.

View	Total (n =)	Training (n =)	Validation (n =)
ME 4-Chamber view	325	260	65
ME Long Axis view	295	236	59
TG LV SAX view	196	156	40
ME AV SAX view	179	143	36
ME Bicaval view	156	124	32
Aortic views	147	117	30
ME 2-Chamber view	93	74	19
ME LAA view	63	50	13
Other	1010	808	202
Total	2464	1968	496

Eight standard TEE view classes were chosen. All TEE videos that did not fall into any of the eight chosen view classes were labeled as “Other.”

TEE, transesophageal echocardiography; ME, mid-esophageal; TG, trans-gastric; LV, left ventricular; SAX, short axis; AV, aortic valve; LAA, left atrial appendage.

**Figure 2:**
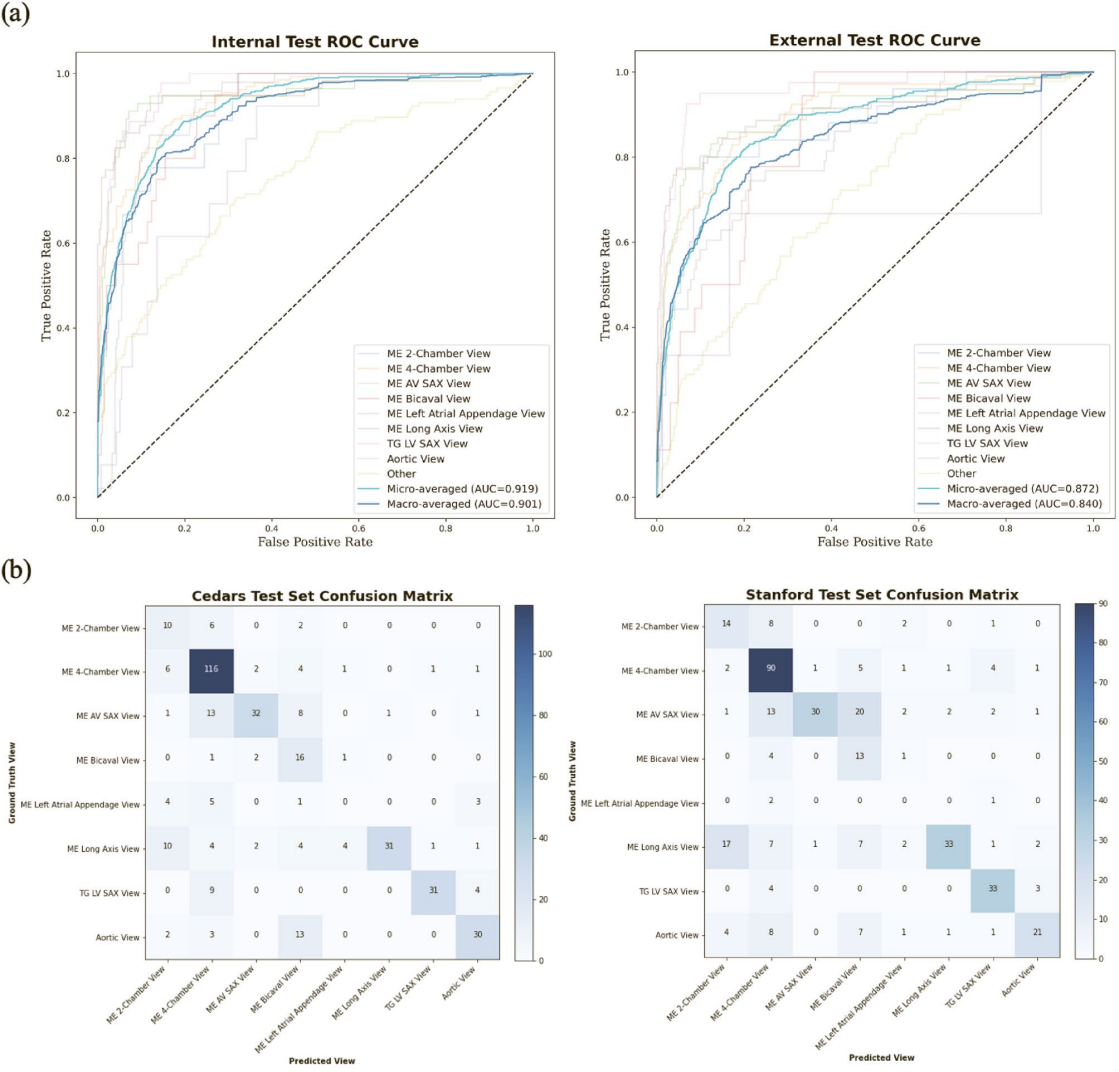
View classification model performance on the internal (CSMC) hold-out test set and the external (SUMC) test set. (**a)** AUC’s for each view class, demonstrating high accuracy (with AUC’s ranging from 0.816 to 0.957). No AUC was able to be calculated for the ME Left Atrial Appendage View in the randomly selected SUMC test set due to low sampling. (**b**) Confusion matrices showing model performance, with views labeled by a board-certified echocardiographer along the vertical axis and views predicted by the deep learning model on the horizontal axis. Numerical values in the matrices and the color intensity of the heatmaps represent the number of images with the indicated ground-truth and model-predicted labels. AUC, area under the receiver operating curve; CSMC, Cedars Sinai Medical Center; SUMC, Stanford University Medical Center; ME, mid-esophageal; AV, aortic valve; SAX, short axis; TG, trans-gastric; LV, left ventricular.

Our view classification model achieved an overall micro-averaged area under the receiver operating curve (AUC) of 0.919 on the hold-out CSMC test set of TEE videos (Fig. 2 and Table 3). Our model showed particularly good performance for the Trans-Gastric Left Ventricular Short Axis View (AUC = 0.971), the Mid-Esophageal Long Axis View (AUC = 0.954), the Mid-Esophageal Aortic Valve Short Axis View (AUC = 0.946), and the Mid-Esophageal 4-Chamber View (AUC = 0.939). The model performance also generalized well externally, achieving a micro-averaged AUC of 0.872 when tested on the 465 never-before-seen TEE videos from SUMC. Our model had similar performance for the Trans-Gastric Left Ventricular Short Axis View (AUC = 0.957), the Mid-Esophageal Long Axis View (AUC = 0.905), the Mid-Esophageal Aortic Valve Short Axis View (AUC = 0.898), and the Mid-Esophageal 4-Chamber View (AUC = 0.902) in the SUMC data set.

**Table 3:** View classification model performance on the internal (CSMC) hold-out test set and the external (SUMC) test set.

View	Internal CSMC Test AUC	External SUMC Test AUC
ME 4-Chamber view	0.939	0.902
ME Long Axis view	0.954	0.905
TG LV SAX view	0.971	0.957
ME AV SAX view	0.946	0.898
ME Bicaval view	0.909	0.846
Aortic views	0.913	0.833
ME 2-Chamber view	0.91	0.87
ME LAA view	0.816	Too few for AUC
Other	0.75	0.706
Overall average AUC	0.919	0.872

AUC’s for each view class and overall micro-averaged AUC’s. No AUC was able to be calculated for the ME Left Atrial Appendage View in the randomly selected SUMC test set due to low sampling.

AUC, area under the receiver operating curve; CSMC, Cedars Sinai Medical Center; SUMC, Stanford University Medical Center.

**Figure 3:**
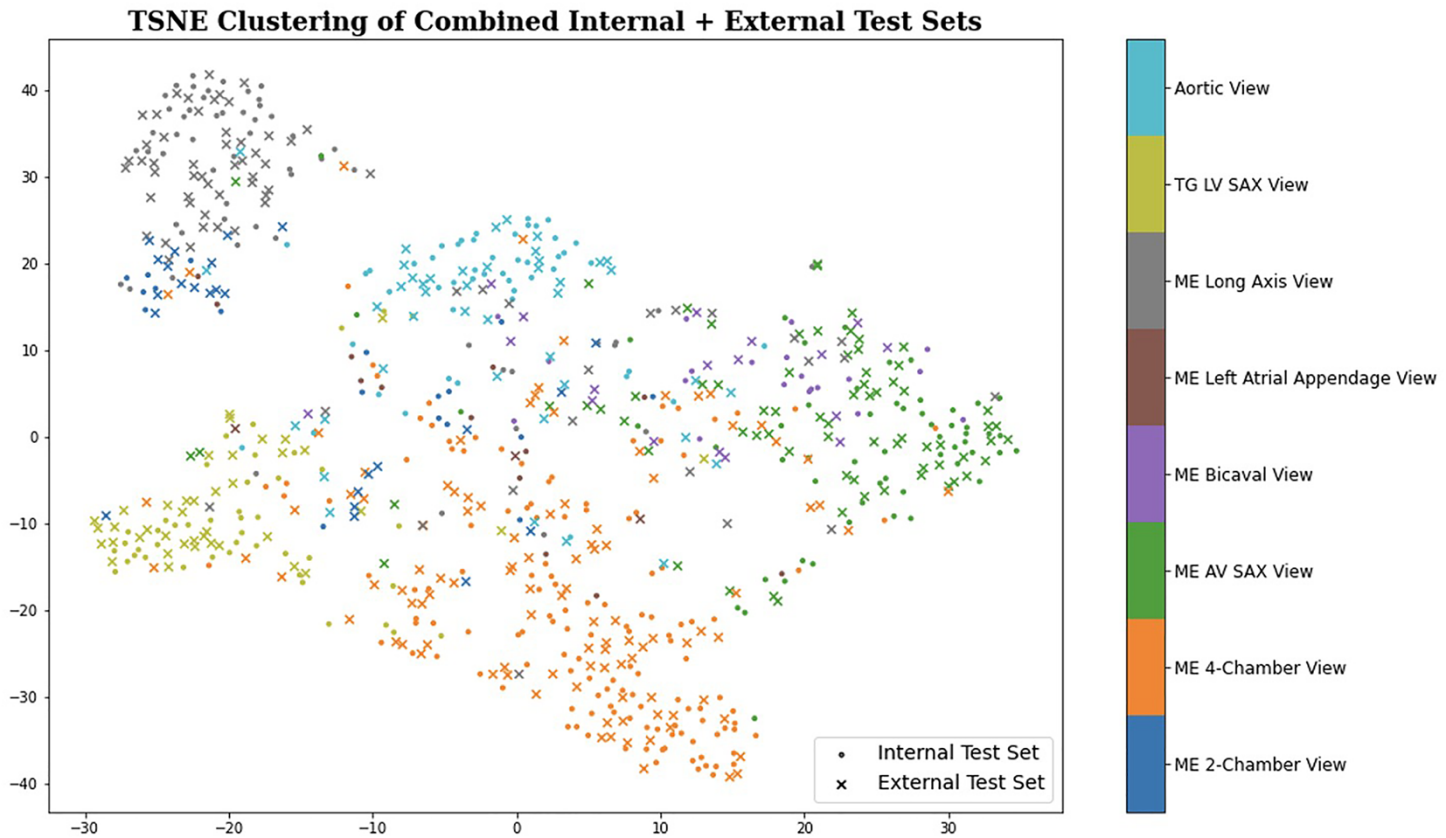
Clustering analysis showing the ability to distinguish among standard TEE views. t-SNE clustering analysis of input images demonstrates that meaningful representations of standard TEE views are clustered appropriately together. In other words, images are sorted into groups that reflect standard TEE classes. Embedding representation is consistent across CSMC and SUMC, suggesting robustness and generalizability of the approach. TEE, transesophageal echocardiography; t-SNE, t-Distributed Stochastic Neighbor Embedding; CSMC, Cedars Sinai Medical Center; SUMC, Stanford University Medical Center.

**Figure 4:**
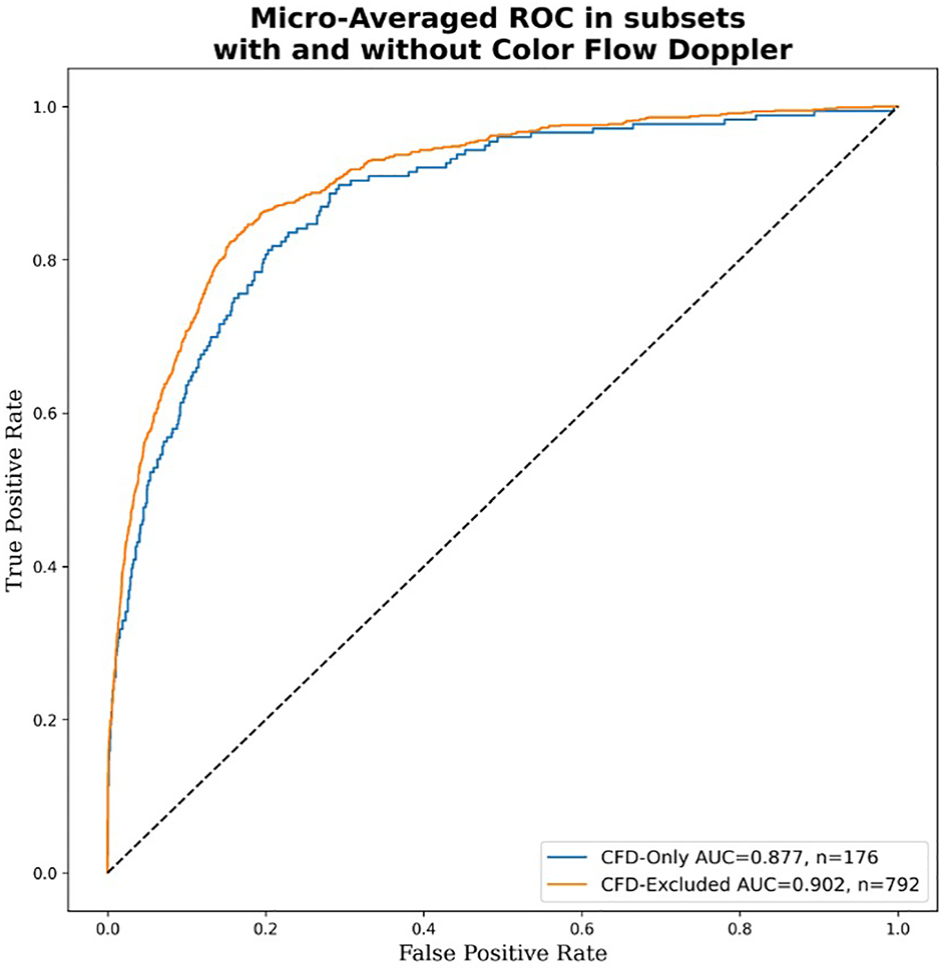
Micro-averaged receiver operating characteristic curves for model predictions in subsets containing all color flow Doppler videos versus no color flow Doppler videos. This evaluation was performed using a combination of the internal and external test sets due to the low prevalence of color flow Doppler videos in our data sets.

Clustering analysis suggests our AI model can identify a meaningful embedding space representing the various TEE views from heterogeneous video input that generalizes across two institutions (Fig. 3). Model performance was similar in standard black-and-white 2D B-Mode TEE videos (micro-averaged AUC = 0.902) and videos incorporating color flow Doppler information (micro-averaged AUC = 0.877) (Fig. 4), the analyses for which were performed on a combination of randomly selected internal and external test videos due to the overall low prevalence of color flow Doppler videos in our data sets.

## Discussion

Our deep learning model was able to classify the eight most commonly used intraoperative and intraprocedural TEE views with high accuracy across a wide range of clinical and echocardiographic characteristics. Our videos included patients undergoing many different types of open cardiac surgery and transcatheter procedures, representing a highly diverse mix of anatomic pathology and differences in practice patterns across two major institutions for cardiology and cardiac surgery. Images also varied with respect to resolution, sizing and focus of the field of view, and the use of color flow Doppler. The model performance was consistently high across the range of findings in both held-out internal and external test data sets, demonstrating the generalizability of our view classifier in real-world clinical contexts.

Our study represents the first application of a machine learning strategy to TEE video image data acquired during the course of standard clinical care for open cardiac surgeries and transcatheter procedures. In prior work, the application of AI strategies to TEE has included focused image segmentation tasks and the automation of specific quantitative measurements. For example, groups such as Carnahan et al.^13^ and Andreassen et al.^14^ demonstrated the ability to identify the mitral valve apparatus from highly curated three- and four-dimensional mid-esophageal-level TEE acquisitions with the mitral valve centered in the images. Tasken et al., was able to automatically quantify the mitral annular plane systolic excursion (MAPSE), using a pipeline that included a view classification task prior to the quantification of MAPSE, highlighting the utility and necessity of TEE view classification for downstream machine learning tasks. Thalappillil et al.^21^ and Li et al.^22^ used quantitative measurements derived from TEE videos, rather than the TEE image data itself, as the input variables or output labels in their machine learning algorithms. Our group is the first to apply machine learning techniques to clinically-acquired intraoperative and intraprocedural TEE image data and the first to add structure to the data contained within these comprehensive clinical TEE exams.

Aside from the limited work that has been done with TEE videos, the large majority of prior AI-driven echocardiography imaged-based studies have focused on TTE videos. For example, it has been demonstrated that machine learning algorithms trained on TTE videos are able to predict standard TTE views^5–8^, identify cardiac structures, estimate cardiac function, make accurate diagnoses, identify phenotypic information that is otherwise not easily recognized by a human observer, and predict clinical outcomes^9–12,23,24^. The major advantage of working with TTE videos over TEE videos for machine learning tasks is that the TTE clinical workflow inherently creates structure for TTE data. As compared to the clinical workflow for TEE imaging, the imaging pipeline for TTE video acquisition, interpretation, and reporting is more standardized and consistent across studies and includes many image annotations and quantitative measurements. The ability to leverage these integrated annotations and quantitative measurements has reduced the need for laborious post hoc image annotation and has facilitated the swift adoption of machine learning for TTE data^9^.

In contrast to the structured nature of TTE data, intraoperative and intraprocedural TEE data are fundamentally more varied and relatively unstructured. The cardiac surgery operating rooms and structural heart disease procedural suites where TEE clinical exams are performed are highly dynamic environments. As a result, the TEE exams performed in these settings often vary in their acquisition sequences and inconsistently include image annotations or quantitative measurements. Moreover, intraoperative and intraprocedural TEE exams are subject to significant variation within and across studies, as they are acquired over the course of significant changes in clinical conditions, including changes cardiac loading, on- versus off-cardiopulmonary bypass (CPB), pre- versus post-surgical intervention, pharmacologic interventions, external cardiac pacing, and the use of other mechanical circulatory support devices. The application of AI-driven strategies to intraoperative and intraprocedural TEE imaging has primarily been limited by the relatively unstructured nature of TEE data. Our present study represents the first attempt at creating structure for clinically-acquired intraoperative and intraprocedural TEE data sets with a machine-learning based view classification algorithm. Additionally, our choice of a video-based model rather than a still image-based model helps to regularize much of the challenging natural variation that occurs with TEE.

Even though multiple AI-driven TTE view classification studies have been conducted in the past^5–7^, the ability to directly apply these tools to TEE data is limited. While TTE and TEE are both ultrasound imaging modalities that capture cardiac structure and function, TTE views and TEE views are not entirely analogous^16,25^. TTE images are acquired from the anterior (trans-thoracic) and left-lateral aspect of the patient, while TEE images are acquired from the posterior (trans-esophageal) aspect of the patient. Additionally, there are differences in probe manipulation and ultrasound beam rotation between the two modalities. The relationship between TTE versus TEE images are more nuanced than a simple one-to-one vertical flip. As a result, the advantages that allowed for the accelerated application of machine learning strategies to structured TTE data could not be automatically applied to TEE data.

The major limitation of our study is the class imbalance present in our data sets. Guidelines established by the American Society of Echocardiography and the Society of Cardiovascular Anesthesiologists identify twenty-eight different TEE views necessary to complete a comprehensive intraoperative multi-plane TEE exam^16^. In actual clinical practice, individual patient factors, anatomic variations and pathology, and time constraints in the cardiac surgery operating rooms and structural heart procedural suites can preclude the acquisition of all twenty-eight views. Oftentimes a comprehensive intraoperative TEE exam will include varying sequences, non-standard views, and multiple missing views. This clinical reality was reflected in our random selection of TEE videos, from both CSMC and SUMC. Many of the twenty-eight standardized TEE views were inconsistently captured and did not yield enough examples for adequate model training, validation, and testing. The most frequently captured views across all randomly selected TEE studies included the ME-4 Chamber View, the ME Long Axis View, the TG Left Ventricular Short Axis View, and the ME Aortic Valve Short Axis View, which is consistent with real-world clinical settings.

While our view classification model performed well across all labeled views, it showed particularly good performance for the views with the most training data and the views with the most visually distinct anatomic features (namely, the ME-4 Chamber View, the ME Long Axis View, the TG Left Ventricular Short Axis View, and the ME Aortic Valve Short Axis View). With respect to clinical significance and plausibility, it is not surprising that the views with most training data are also the views with the most distinct features. The goal of intraoperative echocardiography is to support real-time surgical and procedural decision-making, which requires the efficient acquisition of a complementary set of images that comprehensively captures cardiac structure and function^4^. To this end, the highest yield approach is to focus on a limited set of views that illustrate the relationships among as many significant structures as possible. Collectively, the ME-4 Chamber View, the ME Long Axis View, the TG Left Ventricular Short Axis View, and the ME Aortic Valve Short Axis View efficiently capture the large majority of the information needed by intraoperative and intraprocedural physicians. Therefore, these were the most frequently encountered views in our random selection of TEE videos, and ultimately were the highest performing classes in our view classification model. The performance of our model was the least accurate for the ME Left Atrial Appendage View due to an inadequate number of examples of this view in our random selection of TEE videos. For the future, we will continue to update our view classification model, with a particular focus on increasing the number of labeled examples for rarer views.

**Figure 5:**
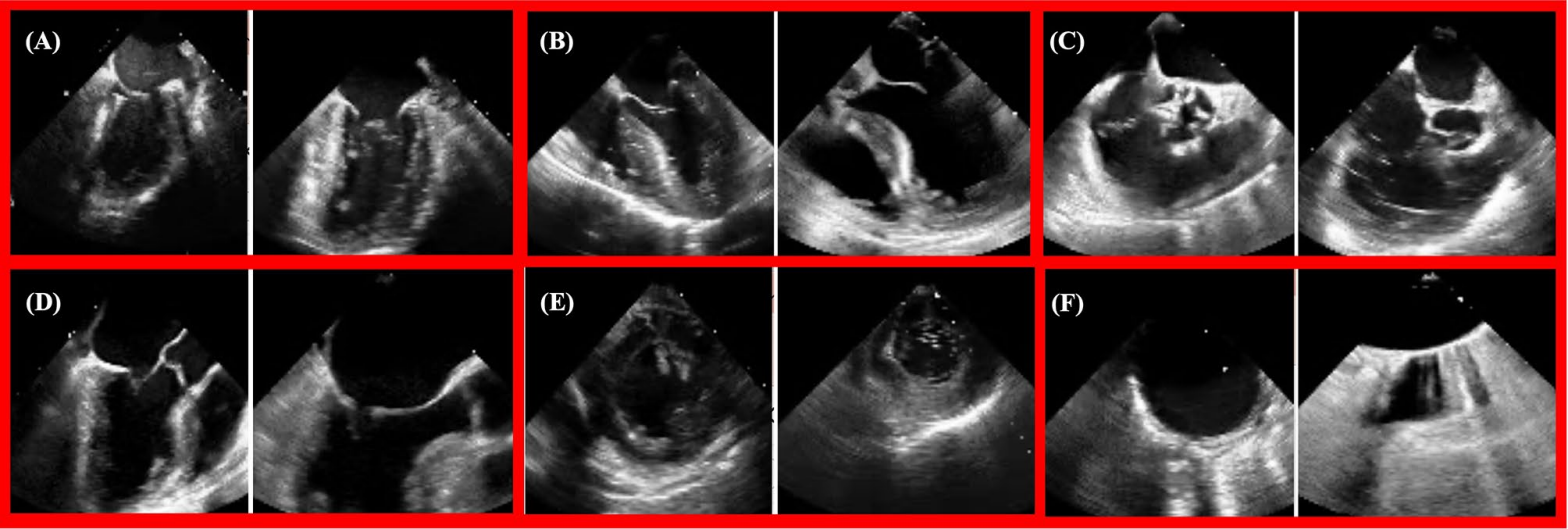
Representation of variation contained within classified views. Six of the eight TEE view class labels represent pooled or generalized categories, reflecting the high degree of anatomical and visual overlap that occurs among the twenty-eight standardized TEE views recommended by the American Society of Echocardiography and the Society of Cardiovascular Anesthesiologists. The images are 2-dimensional still frames sampled from the video data used in model training. (**A**) The “ME 2-Chamber View” class included ME 2-chamber (left) and ME mitral commissural (right) videos. (**B**) The “ME 4-Chamber View” class included ME 4-chamber (left) and ME 5-chamber (right) videos. (**C**) The “ME AV SAX View” class included ME AV SAX (left) and ME RV inflow-outflow (right) videos. (**D**) The “ME Long Axis View” class included ME long axis (left) and ME AV long axis (right) videos. (**E**) The “TG LV SAX View” class included short axis videos of the LV at all levels, such as the mid-papillary (left) and the basal (right) levels. (**F**) The “Aortic View” class included videos of the aorta at all levels, such as the descending thoracic SAX (left) and upper esophageal aortic arch LAX (right) levels. TEE, transesophageal echocardiography; ME, mid-esophageal; AV, aortic valve; SAX, short axis; RV, right ventricular; LV, left ventricular; TG, trans-gastric.

In order to optimize the number of examples that we had per view for model training and to build a view classifier that is reflective of real-world clinical contexts, four of our eight labels (the ME 2-Chamber, ME 4-Chamber, ME AV SAX, ME Long Axis) represent pooled categories. These pooled categories reflect a combination of two standardized views that vary only slightly with regard to omniplane angle, field of view depth, or sector width, but otherwise capture many of the same key structures and anatomic relationships (Fig. 5). The “ME 2-Chamber View” class included ME 2-chamber and ME mitral commissural videos; the “ME 4-Chamber View” class included ME 4-chamber and ME 5-chamber videos; the “ME AV SAX View” class included ME AV SAX and ME right ventricular (RV) inflow-outflow videos; and the “ME Long Axis View” class included ME long axis and ME AV long axis videos. We also chose to generalize two categories (the TG LV SAX and the Aortic Views), in order to increase the sample sizes of these classes (Fig. 5). Any image of the left ventricle in short axis, regardless of level (basal, mid-papillary, or apical), was classified as “Trans-Gastric Left Ventricular Short Axis View.” Similarly, any dedicated image of the aorta, regardless of level or axis orientation, was classified as “Aortic View.” Variation in patient anatomy and dynamic clinical needs often leads to the acquisition of TEE images that do not completely fit the criteria for a specific view class. It is not uncommon to acquire a TEE image that cannot be precisely categorized as a single view but instead falls in between two views. Therefore, combining categories with overlapping anatomic features for our view classification model mirrors real-world clinical practice.

In the present study, we demonstrate that our deep learning model can accurately classify standardized TEE views, which will facilitate further downstream deep learning analyses for intraoperative and intraprocedural TEE imaging. Previous work has already shown that intraoperative TEE imaging actively informs surgical decision-making^26,27^ and is associated with improved clinical outcomes after cardiac surgery^28,29^. The development of AI-driven models based on intraoperative TEE images has the potential to further enhance the value of echocardiography in the perioperative and periprocedural period by improving the ability to diagnose cardiac surgical diseases and complications, diagnose the underlying etiology of varied hemodynamic states, and predict clinical outcomes in the immediate and long-term postoperative periods.

## Conclusion

In summary, we show that an intraoperative and intraprocedural TEE-based deep learning model can accurately identify standardized TEE views, the first step in the AI interpretation of TEE images. Our study represents an important first step towards the automated evaluation of intraoperative and intraprocedural echocardiography imaging and the leveraging of deep learning strategies for the advancement of patient care.

## Data Availability

The data that support the findings of this study are available through Kirsten R. Steffner, MD (ksteffner@stanford.edu). Restrictions apply to the availability of these data, which were used under license for the current study, and so are not publicly available. Data are however available from the corresponding author upon reasonable request and with permission from the Stanford Center for Artificial Intelligence in Medicine & Imaging (aimicenter@stanford.edu).

## Code availability

Our code is available online at https://github.com/echonet/tee-view-classifier.
